# Ultrasound absorption and entropy production in biological tissue: a novel approach to anticancer therapy

**DOI:** 10.1186/1746-1596-1-35

**Published:** 2006-10-06

**Authors:** Liaofu Luo, Joseph Molnar, Hui Ding, Xiaogui Lv, Gabriella Spengler

**Affiliations:** 1 Laboratory of Theoretical Biophysics, Faculty of Science and Technology, Inner Mongolia University, Hohhot, China; 2 Department of Medical Microbiology, Albert Szent-Gyorgyi Medical Center, University of Szeged, Hungary

## Abstract

The entropy production of tumorous cells is higher than that of normal cells, and entropy flow is therefore directed from tumorous toward healthy cells. This results in information concerning the cancer propagating into the surrounding normal tissue. However, ultrasound absorption results in additional entropy production in tissues. The entropy mechanism possibly provides a basis for a novel approach to anticancer therapy through the use of ultrasound irradiation.

Through the calculation of ultrasound-induced entropy production and comparison of the theoretical results with the experimental data on ultrasound absorption in biological tissues, we have demonstrated that ultrasound absorption will increase the entropy in normal tissue more efficiently than in tumorous tissue due to the more acidic nature of the latter. Consequently, the direction of entropy flow between these two kinds of cells may be reversed on exposure to ultrasound.

The higher entropy accumulation of normal cells during ultrasound irradiation may possibly lead to a change in the original direction of entropy flow and avoid the propagation of information on the cancer into the normal tissues. We suggest that low-intensity, low-frequency ultrasound irradiation may be an efficient tool for the therapy of solid tumors.

## Background

Normal tissues contain well-structured, differentiated cells with complex subcellular compartmentization. Cancerous cells are less differentiated than normal cells [1-4]. Entropy is a thermodynamic quantity denoting the level of disorder of a system. The difference between normal and cancerous cells can be studied from the aspect of entropy production [5,6]. In thermodynamics, an isolated system (i.e. one in which there is no exchange of energy and substance with the external world) tends spontaneously and irreversibly toward the state of equilibrium characterized by maximal entropy. This means that the rate of entropy production of an arbitrary system or subsystem is always positive (≥ 0) [7]. The rate of entropy production is a quantity characterizing the thermodynamic property of a system. We have shown that the rate of entropy production in a cancerous cell is higher than that in a normal cell if there is no external energy input [8]. In general, the rate of entropy production in a cell can be expressed in terms of the rates of various irreversible processes and the corresponding "generalized forces". These include [8-10]:

1. the thermal flux driven by a temperature difference;

2. the diffusion current driven by a chemical potential gradient;

3. the rate of a chemical reaction rate driven by a Gibbs energy decrease (affinity);

4. the velocity gradient coupled with viscous stress;

5. the dissipation due to the work performed by an external force field.

The first four terms contribute to a higher rate of entropy production for cancerous cells than for healthy cells, while the case is different for the fifth term. We have demonstrated that an applied electric field produces entropy more efficiently in a normal cell than in a cancerous cell [8]. The difference in entropy production rates between two kinds of cells will influence the direction of entropy flow in biological tissues [10]. Since a cancerous cell is in a high entropy state due to its disordered structure and higher entropy production rate as compared with those of a normal cell, the entropy usually flows from cancerous toward normal tissue, as no external force field is applied. However, application of an electric field may reduce the difference in the rates of entropy production and even change the direction of entropy flow between normal and cancerous cells. This suggests that an external energy input could change the direction of entropy flow due to its possibly larger contribution to entropy accumulation in normal tissue than in cancerous tissue.

For a system with a given distribution of probable states, the Shannon information quantity is defined by I=−∑ipilog⁡2pi
 MathType@MTEF@5@5@+=feaafiart1ev1aaatCvAUfKttLearuWrP9MDH5MBPbIqV92AaeXatLxBI9gBaebbnrfifHhDYfgasaacH8akY=wiFfYdH8Gipec8Eeeu0xXdbba9frFj0=OqFfea0dXdd9vqai=hGuQ8kuc9pgc9s8qqaq=dirpe0xb9q8qiLsFr0=vr0=vr0dc8meaabaqaciaacaGaaeqabaqabeGadaaakeaacqWGjbqscqGH9aqpcqGHsisldaaeqbqaaiabdchaWnaaBaaaleaacqWGPbqAaeqaaaqaaiabdMgaPbqab0GaeyyeIuoakiGbcYgaSjabc+gaVjabcEgaNnaaBaaaleaacqaIYaGmaeqaaOGaemiCaa3aaSbaaSqaaiabdMgaPbqabaaaaa@3E5D@, where *p*_*i *_is the probability of occurrence of the *i*-th chemical, morphological, structural, or physiological state of the cell. For the equiprobable distribution of *N *states, pi=1N
 MathType@MTEF@5@5@+=feaafiart1ev1aaatCvAUfKttLearuWrP9MDH5MBPbIqV92AaeXatLxBI9gBaebbnrfifHhDYfgasaacH8akY=wiFfYdH8Gipec8Eeeu0xXdbba9frFj0=OqFfea0dXdd9vqai=hGuQ8kuc9pgc9s8qqaq=dirpe0xb9q8qiLsFr0=vr0=vr0dc8meaabaqaciaacaGaaeqabaqabeGadaaakeaacqWGWbaCdaWgaaWcbaGaemyAaKgabeaakiabg2da9maalaaabaGaeGymaedabaGaemOta4eaaaaa@32D1@ and we have I=log⁡2N=1ln⁡2ln⁡N
 MathType@MTEF@5@5@+=feaafiart1ev1aaatCvAUfKttLearuWrP9MDH5MBPbIqV92AaeXatLxBI9gBaebbnrfifHhDYfgasaacH8akY=wiFfYdH8Gipec8Eeeu0xXdbba9frFj0=OqFfea0dXdd9vqai=hGuQ8kuc9pgc9s8qqaq=dirpe0xb9q8qiLsFr0=vr0=vr0dc8meaabaqaciaacaGaaeqabaqabeGadaaakeaacqWGjbqscqGH9aqpcyGGSbaBcqGGVbWBcqGGNbWzdaWgaaWcbaGaeGOmaidabeaakiabd6eaojabg2da9maalaaabaGaeGymaedabaGagiiBaWMaeiOBa4MaeGOmaidaaiGbcYgaSjabc6gaUjabd6eaobaa@3EE1@. This information quantity can be compared with the thermodynamic entropy *S *= *k*_*B *_ln *W*, where *W *is the number of microscopic states relating to a given macroscopic thermodynamic state. Thus, the information quantity is proportional to the thermodynamic entropy. More rigorously, since the number of microscopic states *W *is very large, (much larger than the number *N *given in the definition of the Shannon information quantity), we should say that the information quantity is the projection of thermodynamic entropy in the microscopic phase-space to the subspace spanned by *N *macroscopic states [11]. For example, the thermodynamic entropy of a cancerous cell is different from that of a normal cell due to its less ordered structure. Correspondingly, the information inherent in a cancerous cell is different from that in a normal cell. The information quantities in both cancerous and normal cells are described by the above equation, but they have different distributions of {*p*_*i*_}: *p*_*i*_(cancer) ≠ *p*_*i*_(normal) (*i *= 1,...,*N*). We call the information relating to a particular set of {*p*_*i*_} in a cancerous cell {*p*_*i*_(cancer)}, as the information reflecting the particular bias of states in a tumor. The term emphasizes that the information or the distribution {*p*_*i*_(cancer)} in cancer deviates from the normal value. Similarly, the information on a healthy cell is defined by the particular set of {*p*_*i*_} in the healthy cell, {*p*_*i*_(normal)}. By comparison of the definitions of thermodynamic entropy and information quantity, we are able to understand the information flow relating to entropy flow. Since the information quantity is a projection of the thermodynamic entropy, the entropy flow should be the carrier of the information flow. Hence, the entropy flow from a normal to a cancerous cell carries the information on the healthy cell, while the entropy flow in the opposite direction "invasion of malignancy" carries the information on the cancerous cell. This is why we emphasize the meaning of the entropy flow direction.

Study of the rate of entropy production provides opportunities for anticancer therapy. The implication is twofold. On the one hand, we can design approaches by changing the cell parameters or biochemical pathways so as to reduce the rate of entropy production in a cancerous cell. On the other hand, due to the higher entropy in a cancerous cell (and other related factors), the flow of entropy is always in the direction from cancerous to healthy cells, information on the cancerous cell thereby being propagated to the neighbouring healthy cells. If a force field is applied, the additional entropy production will differ between the two kinds of cells. This means a possibility of the reversal of entropy flow and information flow, with a potential positive therapeutic effect. This paper considers ultrasound absorption in cells and demonstrates that the ultrasound-induced rate of entropy production is higher in normal cells than in cancerous cells, which can possibly effectively change the direction of information flow and therefore block the information transfer from cancerous to healthy cells. This would provide a novel approach to anticancer therapy. In recent years, high-intensity focused ultrasound (HIFU) has been used as an effective means of ablating carcinomas [12]. In contrast with HIFU, where high-intensity, high-frequency ultrasound is applied, our proposal concerning ultrasound therapy is based on the thermodynamic principle and entropy production mechanism, and only low-intensity, low-frequency ultrasound is involved.

## Theoretical methods

Our theoretical calculation of ultrasound-induced entropy production in a cell consists in three main steps: calculation of the absorption coefficient of ultrasound in biological tissues; comparison with experimental data and numerical estimation of the ultrasound absorption in biological tissues; and deduction of the ultrasound-induced entropy production in normal and tumorous cells.

The attenuation of an ultrasound wave in a medium is due partly to scattering and partly to absorption. Although ultrasound scattering in normal tissue is appreciably different from that in tumorous tissue because of their structural differences, to discuss entropy production we shall consider absorption only. There are three main mechanisms of ultrasound absorption in biological tissues: absorption due to the shearing motions of medium molecules and viscous forces, heat losses due to conduction, and absorption due to chemical relaxation processes. The former two are often referred to as classical absorption and the third as non-classical absorption. Chemical relaxation occurs when the equilibrium constant of a chemical reaction is affected by pressure changes and/or temperature changes. The ultrasound absorption coefficient α is defined by

*I *= *I*_0 _exp(-α*x*)     (1)

where *x *is the depth of penetration of ultrasound into the biological tissue. Corresponding to the three kinds of absorption mechanism, the absorption coefficient α can be expressed as the sum of three terms:

α = α_η _+ α_κ _+ α_*chm *_    (2)

It has been proved [13,14] that the ultrasound absorption coefficient due to viscous forces is

α_η _= ω^2 ^τ_η_/*c *= 4η ω^2^/3 ρ *c*^3 ^    (3)

and the ultrasound absorption coefficient due to thermal conduction is

α_κ _= ω^2 ^τ_κ_/*c *= ω^2 ^κ_*T *_(γ - 1)/ρ *c*^3 ^*C*_*P *_    (4)

where ρ, η, *C*_*P*_, κ_*T *_and γ are the density of the medium, the coefficient of viscosity of the medium, the constant-pressure specific heat of the medium, the coefficient of thermal conductivity and the ratio of the isobaric and isothermal heat capacities of the medium, respectively. *c *is the velocity of sound, and ω is its angular frequency. The absorption of sound is a relaxation process. Here, τ_η _and τ_κ _in Eqs (3) and (4) are the mechanical and thermal relaxation time, respectively, which are defined through their relation with the absorption coefficient. The total absorption α is related to the equivalent relaxation time Γ [15] as follows:

α=2ωcsin⁡ωΓln⁡γ21+ω2Γ2     (5)
 MathType@MTEF@5@5@+=feaafiart1ev1aaatCvAUfKttLearuWrP9MDH5MBPbIqV92AaeXatLxBI9gBaebbnrfifHhDYfgasaacH8akY=wiFfYdH8Gipec8Eeeu0xXdbba9frFj0=OqFfea0dXdd9vqai=hGuQ8kuc9pgc9s8qqaq=dirpe0xb9q8qiLsFr0=vr0=vr0dc8meaabaqaciaacaGaaeqabaqabeGadaaakeaacqaHXoqycqGH9aqpdaWcaaqaaiabikdaYiabeM8a3bqaaiabdogaJbaacyGGZbWCcqGGPbqAcqGGUbGBdaWcaaqaaiabeM8a3jabfo5ahjGbcYgaSjabc6gaUjabeo7aNbqaaiabgkdaYmaakaaabaGaeyymaeJaey4kaSIaeqyYdC3aaWbaaSqabeaacqGHYaGmaaGccqqHtoWrdaahaaWcbeqaaiabikdaYaaaaeqaaaaakiaaxMaacaWLjaWaaeWaaeaacqaI1aqnaiaawIcacaGLPaaaaaa@4B69@

When the frequency of the ultrasound is low (ωΓ smaller than or close to 1), this leads to

α = ω^2^Γlnγ/*c *    (6)

It should be noted that the equivalent relaxation time Γ in ref. [15] is defined differently from the relaxation times τ_η _and τ_κ _in Eqs (3) and (4).

Let us now consider numerical estimates of sound absorption in biological tissues. Taking η = 0.015, ω = 6.28 × 10^6 ^(corresponding to 1 MHz), ρ = 1, *c *= 1.56 × 10^5^, γ - 1 = 7 × 10^-4^, *C*_*p *_= 4.18 × 10^7^, κ_*T *_= 5.88 × 10^4^

(all in CGS units) and using Eqs (3) and (4), we have

α_η _= 2 × 10^-4 ^cm^-1 ^    (7)

α_κ _= 1.0 × 10^-8 ^cm^-1 ^    (8)

Next, by using

lnγ ≈ γ - 1 = 7 × 10^-4^, Γ = 0.91 × 10^-6 ^[15], *c *= 1.56 × 10^5^, ω = 6.28 × 10^6 ^(all in CGS units) and Eq. (6), we estimate the value of total absorption α as

α = 1.61 × 10^-1 ^cm^-1 ^    (9)

Comparison of Eq. (7), Eq. (8) and Eq. (9) reveals that

α_η _<<α and α_κ _<<α     (10)

We therefore conclude that the most important contribution to the low-frequency ultrasound absorption (α) is that of chemical relaxation (α_*chm*_).

Experimental data on the equivalent relaxation time Γ in biological tissues are presented in Table 1.

**Table 1:** Relaxation time Γ in various human biological tissues

kidney	heart	testis	brain	liver	tendon	blood	amniotic fluid
0.91 μ*s*	0.64 μ*s*	0.44 μ*s*	0.15 μ*s*	0.13 μ*s*	0.10 μ*s*	0.06 μ*s*	0.02 μ*s*

The experimental data are taken from [15].

These data show that the sound absorption due to chemical relaxation differs greatly from tissue to tissue.

Another important point is the strong dependence of ultrasound absorption on the pH level. It has been demonstrated that α increases monotonously with pH in the range from 6 to 12, e.g. α = 0.1 dB/cm at pH = 6.4 to α = 0.2 dB/cm at pH = 12 (beef liver supernatant, at 1 MHz) [16]. This is understandable since chemical relaxation makes the main contribution to sound absorption, and the equilibrium constant of a chemical reaction is dependent on the pH of the cellular environment.

Ultrasound absorption in biological tissues leads to additional entropy production in the cells. After absorption the sound energy is transformed into heat. The rate of heat transfer to a cell during ultrasound irradiation is

dQdt=α LAW     (11)
 MathType@MTEF@5@5@+=feaafiart1ev1aaatCvAUfKttLearuWrP9MDH5MBPbIqV92AaeXatLxBI9gBaebbnrfifHhDYfgasaacH8akY=wiFfYdH8Gipec8Eeeu0xXdbba9frFj0=OqFfea0dXdd9vqai=hGuQ8kuc9pgc9s8qqaq=dirpe0xb9q8qiLsFr0=vr0=vr0dc8meaabaqaciaacaGaaeqabaqabeGadaaakeaadaWcaaqaaiabdsgaKjabdgfarbqaaiabdsgaKjabdsha0baacqGH9aqpcqaHXoqycqqGGaaicqWGmbatcqWGbbqqcqWGxbWvcaWLjaGaaCzcamaabmaabaGaeGymaeJaeGymaedacaGLOaGaayzkaaaaaa@3D76@

(where *W *is the ultrasound intensity, and *L *and *A *are the cell diameter and average cross-section respectively). This transfer causes changes the internal energy and entropy of the cell. From thermodynamics, the increase in the internal energy is

ΔU=∫cvdT     (12)
 MathType@MTEF@5@5@+=feaafiart1ev1aqatCvAUfKttLearuWrP9MDH5MBPbIqV92AaeXatLxBI9gBaebbnrfifHhDYfgasaacH8akY=wiFfYdH8Gipec8Eeeu0xXdbba9frFj0=OqFfea0dXdd9vqai=hGuQ8kuc9pgc9s8qqaq=dirpe0xb9q8qiLsFr0=vr0=vr0dc8meaabaqaciaacaGaaeqabaqabeGadaaakeaacqqHuoarcqWGvbqvcqGH9aqpdaWdbaqaaiabdogaJnaaBaaaleaacqWG2bGDaeqaaaqabeqaniabgUIiYdGccqWGKbazcqWGubavcaWLjaGaaCzcamaabmaabaGaeGymaeJaeGOmaidacaGLOaGaayzkaaaaaa@3C67@

(*c*_*V *_is the isothermal heat capacity and *T *is absolute temperature). In the event of low-intensity ultrasound irradiation, the change in *T *is small and can be neglected; hence Δ*U *= 0. The rate of production of thermodynamic entropy in a cell is

ΔS=∫dQT=1TΔQ     (13)
 MathType@MTEF@5@5@+=feaafiart1ev1aaatCvAUfKttLearuWrP9MDH5MBPbIqV92AaeXatLxBI9gBaebbnrfifHhDYfgasaacH8akY=wiFfYdH8Gipec8Eeeu0xXdbba9frFj0=OqFfea0dXdd9vqai=hGuQ8kuc9pgc9s8qqaq=dirpe0xb9q8qiLsFr0=vr0=vr0dc8meaabaqaciaacaGaaeqabaqabeGadaaakeaacqqHuoarcqWGtbWucqGH9aqpdaWdbaqaamaalaaabaGaemizaqMaemyuaefabaGaemivaqfaaaWcbeqab0Gaey4kIipakiabg2da9maalaaabaGaeGymaedabaGaemivaqfaaiabfs5aejabdgfarjaaxMaacaWLjaWaaeWaaeaacqaIXaqmcqaIZaWmaiaawIcacaGLPaaaaaa@4082@

(deduced by assuming constant temperature in the absorption process). From Eqs (11) and (13), we obtain the entropy production rate (EPR) during ultrasound irradiation of a cell:

*EPR *= α*LAW*/*T *    (14)

If we take α = 0.23 × 10^-1 ^(absorption coefficient for liver at 1 MHz ultrasound [15,16]) *L *= 0.5 × 10^-2^, *A *= π*L*^2^/8, W = 1 watt/cm^2 ^= 10^7 ^(presumed arbitrarily) and *T *= 310 (all in CGS units) the numerical estimate of the rate of entropy production of a cell due to low-frequency (1 MHz) low-intensity (1 watt/cm^2^) ultrasound absorption gives

EPR = 0.38 × 10^-4 ^erg/degree/s     (15)

Let us consider the difference in EPR between cancerous and normal cells. Let us suppose that the protein composition is nearly the same in cancerous and normal cells in a given tissue. This assumption is acceptable for many types of cancer. With this assumption, the difference in ultrasound absorption between the two kinds of cells stems mainly from the difference in their pH values. The pH for a normal cell ranges from 7.35 to 7.45, while for a cancerous cell it ranges from 6.85 to 6.95. If it is considered that the ultrasound absorption coefficient α increases monotonously with pH in the range from 6 to 8, we can deduce that a healthy cell exhibits higher ultrasound absorption than a cancerous cell. Assuming that the relative difference in α between these two cells is *P *= 0.1, we estimate that

EPR (normal) - EPR (cancer) = 0.38 × 10^-4 ^*P *= 0.38 × 10^-5 ^erg/degree/s     (16)

## Results and discussion

1. Comparison of the mechanical and thermal mechanisms of the absorption of ultrasound with the experimental data indicates that chemical relaxation is the main mechanism of ultrasound absorption in biological tissues. The ultrasound absorption coefficients due to viscous force and thermal conduction are given by Eqs (3) and (4). They are related to physical parameters of the medium such as viscosity, thermal conductivity, etc., but independent of the morphological, structural and physiological properties of the cells. Thus, the relevant conclusions deduced in this paper are valid generally, and no special assumption concerning the morphology or structure of the cells is required.

2. The absorption of ultrasound in a biological medium is strongly dependent on the nature of the tissue. The relaxation time measured experimentally ranges from about 0.1 to 1 μ*s*. Accordingly, the EPR due to ultrasound absorption is (0.3 to 3) × 10^-4 ^erg/degree/s for 1 MHz 1 watt/cm^2 ^ultrasound. In previous work [8], we estimated that, for a cell in which no external force field is applied, EPR is 10^-7 ^erg/degree/s or lower. Thus, one per cent of 1 watt/cm^2 ^of ultrasound irradiation can effectively induce entropy production in a cell which is comparable with the case of no field applied.

3. In consequence of the chemical absorption mechanism, the ultrasound absorption and in turn the entropy production are sensitively dependent on the pH of the biological medium. Since ultrasound absorption increases monotonously with pH in a wide range, we conclude that a healthy cell displays a higher entropy production than cancerous cell because of the latter being more acidic. We estimate the difference in EPR between the two kinds of cells to be about (0.3 to 3) × 10^-5 ^erg/degree/s under 1 MHz 1 watt/cm^2 ^ultrasound. Hence, several per cent of 1 watt/cm^2 ^of ultrasound irradiation is sufficient to reverse the direction of entropy flow. Such a reversal of entropy flow would prevent the propagation of information on the cancerous tissue into the normal tissue. We suggest that this entropy mechanism could possibly provide the basis for a novel approach to anticancer therapy.

4. Ultrasound absorption increases with the square of frequency (Eq, (6)). The propagation of high-frequency, high-intensity ultrasound in biological tissue would cause too rapid absorption. Following its absorption in biological tissue, the energy of the ultrasound wave is transformed into thermal energy. When the thermal energy accumulation in a cell is so rapid that it cannot be effectively dissipated into its environment, the temperature and internal energy of the cell rise. The damaging effect of high temperature on cells is a favourable factor in certain surgical operations. Moreover, due to the structural changes in them, the blood supply of some kinds of cancerous cells is insufficient. For example, the blood circulation in some tumours is only 2% to 15% of that in the surrounding healthy tissue. This makes the temperature of a tumour explicitly higher than that of the surrounding healthy tissue during strong ultrasound irradiation [17]. The above result is again favourable for HIFU therapy. However, for the purpose of anticancer therapy we suggest the use of low-intensity, low-frequency ultrasound irradiation since in this case the ultrasound is only weakly absorbed in the biological tissue and the induced temperature increase can be neglected, so that the effect of entropy production on the non-damaged cells can be studied independently of a temperature effect.

The application of ultrasound in anticancer therapy has a long history. In the 1940s, the clinical application of ultrasound revealed curative possibilities. In 1944, when Horvath first used ultrasound in human tumor treatment, after 3 days of operation at 800 kHz, he found that the cancer cells had disintegrated. Horvath later conveyed the ultrasound wave to water to irradiate the tumor and gained a favorable effect (1946, 1947). Several illustrations of the cure of skin cancer by ultrasound have been reported by Demmel (1948), Kemper (1948) and Woeber (1949, 1950) [18]. In the 1990s, HIFU was applied as an effective technique for the ablation of carcinomas [12,17]. Although HIFU affords effective and relatively safe treatment, several complications have been observed following the treatment of certain tumour types with this modality [19]. In the meantime, ultrasound at lower frequencies has been used to enhance drug penetration into the tissues by cavitation [17]. Differently from previous investigations, the entropy mechanism proposed in this article provides a novel approach to the anticancer application of ultrasound. In fact, the success of low-frequency, low-intensity ultrasound therapy in various cases of cancer has been reported [18]. This could perhaps be explained by the entropy mechanism proposed here.

## Conclusion

In consequence of the higher rate of entropy production of normal cells during ultrasound irradiation as compared with that of cancerous cells the original direction of entropy flow from the cancerous to the surrounding normal tissue should be reversed. Since the information quantity is the projection of the thermodynamic entropy from the microscopic phase-space to the subspace spanned by the macroscopic states, the entropy flow is the carrier of the information flow. While the entropy flow from the tumorous to the normal cells carries the information relating to the cancerous cells, the entropy flow in the opposite direction carries the information on the healthy tissue. Accordingly, the reversal of the entropy flow direction in the biological tissues during low-frequency, low-intensity ultrasound irradiation can prevent the propagation of information from cancerous cells. We suggest that this entropy mechanism could possibly provide a basis for a novel approach to anticancer therapy.

## Competing interests

None

## Authors' contributions

LL: proposed the idea concerning changing the entropy flow direction by means of ultrasound irradiation, carried out the calculation of ultrasound – induced entropy production, and wrote the original version of the manuscript.

JM: conceived the thermodynamic approach to anticancer effects and entropy dissipation, and participated in the writing of the manuscript.

HD: collected the related literature on ultrasound studies and carried out some of the calculations.

XL: collected the data on ultrasound absorption in biological tissues a and carried out some of the calculations together with,

GS, who participated in the writing of the manuscript and performed the work relating to the online submission on the manuscript.

All authors have read and approved the final manuscript.
